# Sedentary behaviors, physical activity behaviors, and body fat in 6-year-old children: the Generation R Study

**DOI:** 10.1186/s12966-014-0096-x

**Published:** 2014-08-15

**Authors:** Anne I Wijtzes, Selma H Bouthoorn, Wilma Jansen, Oscar H Franco, Albert Hofman, Vincent WV Jaddoe, Hein Raat

**Affiliations:** The Generation R Study Group, Erasmus Medical Center, Rotterdam, The Netherlands; Department of Public Health, Erasmus Medical Center, University Medical Center, P.O. Box 2040, 3000 CA Rotterdam, The Netherlands; Department of Social Development, Rotterdam, The Netherlands; Department of Epidemiology, Erasmus Medical Center, Rotterdam, The Netherlands; Department of Pediatrics, Erasmus Medical Center, Rotterdam, The Netherlands

**Keywords:** Body fat, Overweight, Lifestyle, Physical activity, Sedentary, Children

## Abstract

**Background:**

Childhood overweight and obesity is a major public health concern. Knowledge on modifiable risk factors is needed to design effective intervention programs. This study aimed to assess associations of children’s sedentary behaviors (television viewing and computer game use) and physical activity behaviors (sports participation, outdoor play, and active transport to/from school) with three indicators of body fat, i.e., percent fat mass, body mass index (BMI) standard deviation scores, and weight status (normal weight, overweight).

**Methods:**

Cross-sectional data from 5913 6-year-old ethnically diverse children were analyzed. Children’s weight and height were objectively measured and converted to BMI. Weight status was defined according to age- and sex-specific cut-off points of the International Obesity Task Force. BMI standard deviation scores were created, based on Dutch reference growth curves. Fat mass was measured my dual-energy X-ray absorptiometry (DXA). Sedentary and physical activity behaviors were assessed by parent-reported questionnaires. Series of logistic and linear regression analyses were performed, controlling for confounders (i.e., socio-demographic factors, family lifestyle factors, and other sedentary behaviors and physical activity behaviors).

**Results:**

Sports participation was inversely associated with fat mass (p < 0.001), even after adjustment for socio-demographic factors, family lifestyle factors, and other sedentary behaviors and physical activity behaviors. No other independent associations were observed.

**Conclusions:**

The results of this study indicate that sports participation is inversely associated with percent body fat among ethnically diverse 6-year-old children. More research in varied populations including objective measurements and longitudinal designs are needed to confirm these current results.

## Introduction

Childhood overweight and obesity is currently one of the major challenges in public health [1]. Childhood overweight is associated with a wide range of adverse physical and psychological outcomes including asthma, hypertension, type 2 diabetes, sleeping disorders, and low self-esteem [1,2]. Furthermore, childhood overweight has been shown to adversely affect cardiovascular morbidity and premature mortality in adulthood, either through tracking of overweight into adulthood or through independent effects of childhood overweight [3]. In order to tackle the current childhood overweight epidemic, insight into the underlying modifiable determinants is essential.

The increased prevalence of childhood overweight has been previously attributed to reductions in physical activity and increases in sedentary behaviors among children [4,5]. Television viewing especially is highly prevalent among young children [6], and both cross-sectional and longitudinal studies support the association between children’s television viewing time and overweight [7]. The evidence for a similar association has been less consistent for computer use, possibly due to the more active nature of this activity [7]. Studies on cross-sectional associations between children’s physical activity and overweight generally support the hypothesis that physical activity is protective against childhood overweight and obesity [8]; however, there is inconsistent evidence on the longitudinal associations between physical activity and childhood adiposity [9-11].

Only few studies in children have used dual energy X-ray absorptiometry (DXA) instead of proxy measures for body fat such as skinfolds and body mass index (BMI) [12-17]. Furthermore, earlier studies have been inconsistent in adjusting for dietary behaviors known to affect childhood overweight, such as consumption of breakfast and sugar-sweetened beverages [12-14,16]. Therefore, confounding by these factors cannot be ruled out. In this study we aimed to assess the independent associations of children’s sedentary behaviors and physical activity behaviors with three indicators of body fat, i.e., percent fat mass, body mass index (BMI), and weight status in 6-year-old children. This study used data from the Generation R Study, a large, multiethnic, birth cohort in Rotterdam, The Netherlands.

## Methods

### Study design

This cross-sectional study was embedded in the Generation R Study, a population-based cohort study from fetal life onwards. The Generation R Study was designed to identify early environmental and genetic determinants of growth, development and health, and has been described previously in detail [18]. The study was conducted in accordance with the guidelines proposed in the World Medical Association Declaration of Helsinki and has been approved by the Medical Ethical Committee at Erasmus MC, University Medical Center Rotterdam. Written informed consent was obtained from all parents.

### Study population

**Figure 1:**
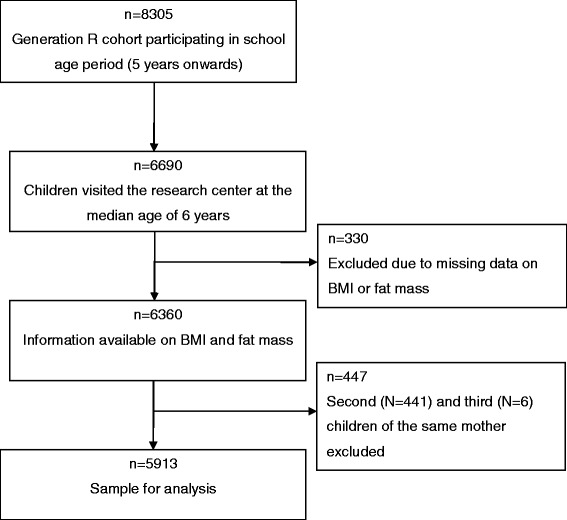
**Flowchart of participants.**

Invitations to participate in the study were made to all pregnant women who had an expected delivery date between April 2002 and January 2006 and who lived in the study area (Rotterdam, The Netherlands) at time of delivery. In total, 8305 children from the original 9749 known live born children of the Generation R cohort participate in the school aged period (5 years onwards). At the age of 6 years, participating children and their mothers were invited to a well-equipped and dedicated research center in the Erasmus Medical Center- Sophia’s Children’s Hospital. Of those, 6690 children visited the research center where information on body composition of the children was collected [18]. We excluded participants with missing information on BMI or fat mass (n = 330). To avoid clustering of data, we furthermore excluded second (n = 441) and third children (n = 6) of the same mother, leaving a study population of 5913 participants (Figure 1).

### Indicators of body fat

Children’s weight and height were measured in the research center using standardized procedures and devices. Weight was measured in lightweight clothes and without shoes with a mechanical personal scale (SECA) and height was measured using a Harpenden stadiometer (Holtain Limited) in standing position, both of which were calibrated on a regular basis. BMI (kg/m^2^) was calculated as [weight(kg)]/[height(m)]^2^. Using the Growth Analyzer program (Growth Analyzer 3.0, Dutch Growth Research Foundation, Rotterdam, The Netherlands.), standard deviation scores (SDS) adjusted for age and gender were constructed based on Dutch reference growth curves [19]. Children’s weight status was defined according to age- and sex-specific cut-off points proposed by the International Obesity Task Force [20]. DXA scans (iDXA; General Electric, formerly Lunar Corp., Madison, WI) were used to obtain percent fat mass of the children. The DXA scans provide measurements of bone and soft tissue for the total body and sub regions, including bone mineral content (g), fat mass (g), and lean mass (g). Percent fat mass was calculated as 100 % × [total body fat mass(g)]/[total body mass(fat mass + lean mass + bone mass of total body)(g)]. Children were scanned in a supine position with their feet together in a neutral position and hands flat by their sides. All DXA scans were obtained using the same device and software (enCORE2010) and were performed by well-trained and certified research staff.

### Sedentary behaviors and physical activity behaviors

Sedentary behaviors and physical activity behaviors were assessed by parent-reported questionnaire when the child was 6 years old. Key sedentary behaviors included television viewing (including video/DVD) and computer game use (including video games). For both of the variables, frequency (i.e., number of days) and duration (i.e., minutes) were asked for weekdays and weekend days separately. These variables were combined to estimate daily television viewing by using the following formula: daily use = {[(days per week) * (hours on a weekday)] + [(days per weekend) * (hours on a weekend day)]}/7. Average daily television viewing was then dichotomized into ≥2 hours/day versus <2 hours/day, based on current recommendations on screen-based entertainment for young children [6,21,22]. Average daily computer game use was dichotomized into ≥1 hour/day versus <1 hour/day, since only a small percent of children used the computer (games) for 1 or more hours per day.

Key physical activity behaviors included outdoor play, sports participation, and active transport to/from school. Similar to television viewing and computer game use, frequency and duration of outdoor play were assessed for weekdays and weekend days separately and combined to estimate daily outdoor play using the same formula (i.e., estimated total weekly hours divided by 7 days). Daily outdoor play was dichotomized into <1 hour/day versus ≥1 hour/day according to physical activity guidelines stating that children should acquire at least one hour of moderate to vigorous physical activity (MVPA) per day [23-25]. For active transport, number of days on which the children walked to/from school (0–5 days) and number of days children cycled to/from school (0–5 days) were assessed. Days per week of active transport was calculated by adding these numbers and dichotomized into < 5 days/week versus 5 days/week. For sports participation (no, yes), the following question was used: “Does your child take part in sports (for example, soccer, judo, gymnastics, jazz ballet, tennis, etc.?)”. School sports activities such as physical education lessons and swimming lessons were not included in this question.

### Potential confounders

The following risk factors for childhood overweight were selected as potential confounders based on previous literature [26-28]: child’s sex, age, and ethnic background, family socioeconomic position (SEP), parental BMI, and children’s dietary behaviors. Indicators of family SEP were assessed by questionnaire when the child was 6 years old and included maternal educational level (highest level attained), maternal employment status (paid job, no paid job), household income (<€1600/$2166 per month, ≥€1600/$2166 per month), and single parenthood (single parent, two parents). The Dutch Standard Classification of Education was used to categorize four levels of education: low (no education, primary school, or three years or less general secondary school), mid-low (more than three years general secondary school), mid-high (higher vocational training) and high (university or PhD degree) [29]. In accordance with Statistics Netherlands, ethnic background of the child was defined according to country of birth of the child’s parents [30]. Children with both parents born in the Netherlands were considered native Dutch, children with at least one parent born in Europe (Turkey excluded), North-America, Oceania, Indonesia, and Japan were assigned an other-Western ethnic background, and children with at least one parent born in another country were assigned a non-Western ethnic background [30]. Maternal BMI was calculated on the basis of self-reported pre-pregnancy weight and measured height at enrollment (kg/m^2^). Maternal BMI was assessed again when the child was 6 years old. The correlation between these two variables was high (Pearson’s correlation coefficient: 0.83; p < 0.001). Maternal pre-pregnancy was used in the analyses because data were less often missing for this variable. Paternal BMI was calculated on the basis of measured weight and height at enrollment. Children’s dietary behaviors included breakfast skipping (yes, no), consumption of sugar-containing beverages (e.g., soft drinks, fruit juices, lemonade, and sweetened milk products such as chocolate milk) (into ≥ 3 glasses/day, <3 glasses per day), and consumption of high-calorie snacks (e.g., sweets, potato chips, chocolate bars, ice cream) (≥2 times/day, < 2 times/day) [28].

### Statistical analyses

Descriptive statistics were used to characterize the total study population and children with and without overweight. Differences in variables between children with normal weight and children with overweight (including obesity) were evaluated using ANOVAs or Kruskal-Wallis tests for continuous variables and Chi-square tests for categorical variables. The correlation between BMI SDS and fat mass was assessed by Pearson’s correlation coefficient. Series of multiple logistic and linear regression models were used to assess the associations of sedentary behaviors and physical activity behaviors with overweight (including obesity), BMI SDS, and percent fat mass, respectively. Associations between the behaviors and each of the three indicators were first assessed in crude models. The second set of models (model 1), was adjusted for socio-demographic factors. The third set of models (model 2) was additionally adjusted for family lifestyle factors (i.e., parental BMI and children’s dietary behaviors). Sedentary behaviors and physical activity behaviors associated with the outcomes in model 2 with a p value <0.20 were simultaneously entered into fully adjusted models (model 3) to assess their independent associations with the outcomes. Multinomial logistic regression analyses, using the same models, were performed to assess associations with overweight and obesity separately. Previous studies conducted in older school aged children (i.e. ≥ 8 years) have shown different effects for boys and girls [5,31,32]. However, less is known about potential effect modification by child’s sex in younger children [28,33]. Therefore, interactions with child’s sex were explored. When significant, stratified analyses were performed. To handle missing data, multiple imputation was applied [34]. Missing data ranged between 0% for child’s age, sex, and outcome variables, and 31.3% for paternal BMI (Table 1). Five imputed datasets were generated using a fully conditional specified model, thus taking into account the uncertainty of the imputed values. Pooled estimates from these five imputed datasets were used to report ORs, beta’s, and their 95% confidence intervals (CIs). Imputations were based on the relationships between all the variables included in this study. All analyses were conducted in 2012 with Statistical Package for Social Sciences (SPSS) version 20.0 for Windows (SPSS Inc., Chicago, IL, USA). A significance level of p < 0.05 was used to indicate significant associations.

**Table 1:** **Descriptive characteristics of the total study population and according to child’s weight status (n = 5913)**

**Characteristics**		**Total (n = 5913)**	**Normal weight (n = 4870) (82.4%)**	**Overweight (including obesity) (n = 1043) (17.6%)**	p-Value*
*Socio-demographic characteristics*					
Child’s sex	Girl (%)	50.3	48.5	58.7	<0.001
Child’s age (years)	(Median, 90% range)	6.0 (5.7-7.4)	6.0 (5.7-7.2)	6.1 (5.8-7.8)	<0.001
Child’s ethnic background	Dutch (%)	56.1	59.8	38.9	<0.001
	Other-western (%)	9.1	9.2	8.4	
	Non-western (%)	34.8	31.0	52.7	
Maternal educational level	High (%)	27.2	29.5	15.6	<0.001
	Mid-high (%)	27.2	28.3	21.7	
	Mid-low (%)	32.2	30.7	39.7	
	Low (%)	13.4	11.5	23.0	
Maternal employment status	No paid job (%)	25.2	23.7	33.3	<0.001
Household income	<€1600/$2166 per month (%)	17.6	16.1	25.7	<0.001
Single parenthood	Single parent (%)	14.9	14.0	19.6	<0.001
*Parental anthropometric measures*					
Maternal pre-pregnancy BMI (kg/m^2^)	(Mean, SD)	23.6 (4.2)	23.1 (3.8)	25.9 (5.2)	<0.001
Paternal BMI (kg/m^2^)	(Mean, SD)	25.3 (3.5)	24.9 (3.2)	27.1 (4.1)	<0.001
*Child anthropometric measures*					
Weight (kg)	(Mean, SD)	23.3 (4.3)	22.1 (2.8)	29.1 (5.1)	<0.001
Height (cm)	(Mean, SD)	119.6 (6.0)	119.0 (5.7)	122.3 (6.6)	<0.001
BMI SDS	(Mean, SD)	0.3 (0.9)	−0.0 (0.7)	1.7 (0.6)	<0.001
Fat mass (%)	(Mean, SD)	25.0 (5.7)	23.3 (4.1)	32.7 (5.6)	<0.001
*Child sedentary behaviors*					
Television viewing	≥ 2 hours/day (%)	19.9	18.5	27.6	<0.001
Computer game use	≥ 1 hour/day (%)	7.6	7.5	8.2	0.53
*Child physical activity behaviors*					
Outdoor play	< 1 hour/day (%)	34.5	33.4	41.0	<0.001
Sport participation	No (%)	55.6	54.5	60.8	<0.01
Active transport	<5 days/week (%)	57.4	58.9	49.3	<0.001

Table is based on non-imputed dataset.

Missings were 0 for child’s sex, 0 for child’s age, 155 (2.6%) for child’s ethnicity, 915 (15.5%) for maternal educational level, 1186 (20.1%) for maternal employment status, 1196 (20.2%) for household income, 892 (15.1%) for single parenthood, 1500 (25.4%) for maternal pre-pregnancy BMI, 1852 (31.3%) for paternal BMI, 1340 (22.7%) for TV viewing, 1357 (22.9%) for computer game use, 1765 (29.8%) for playing outside, 938 (15.9%) for sport participation, and 1273 (21.5%) for active transport to/from school.

*Differences between normal weight and overweight (including obesity) children were evaluated using ANOVA and Kruskal-Wallis tests for continuous variables, and Chi-square tests for categorical variables.

## Results

Table 1 shows characteristics of the study population and according to child’s weight status. Nearly 20% of all children were overweight (including obesity). One quarter of overweight children were obese. Overweight children were older (p < 0.001), more often girls (p < 0.001), more often of Non-Western ethnicity (p < 0.001), and more often of low family SEP (all p < 0.001) compared with normal weight children. Overweight children had a higher percent fat mass than normal weight children (p < 0.001). Pearson’s correlation coefficient for the correlation between BMI SDS and percent fat mass was moderate (r = 0.65; p < 0.001).

We did not find significant interactions of child’s sex with any of the outcomes; therefore results are presented for the total study population. Television viewing was significantly positively associated with all three outcomes in the crude models (all p < 0.001) (Tables 2, 3, and 4). However, these associations disappeared after adjustment for socio-demographic factors (for outcomes weight status and BMI-SDS) or following additional adjustment for family lifestyle factors (for outcome percent fat mass). Computer game use was positively associated with BMI SDS in the crude model only (p < 0.05) (Table 3). Outdoor play was significantly inversely associated with BMI SDS and weight status (both p < 0.001) and fat mass (p < 0.01) in the crude models, but these associations disappeared after adjustment for socio-demographic factors (for outcome percent fat mass) or additional adjustment for family lifestyle factors (for outcomes weight status and BMI SDS). Sports participation was significantly inversely associated with percent fat mass in all models, including the fully adjusted model (all p < 0.001) (Table 4). Sports participation was also positively associated with children’s weight status, but only in the crude model (p < 0.01) (Table 2). Active transport was inversely associated with all three outcomes in the crude models (all p < 0.01), but these associations attenuated following correction for socio-demographic factors. Results from the multinomial analyses presenting associations with overweight and obesity separately were highly similar (Table 5).

**Table 2:** **Associations of sedentary behaviors and physical activity behaviors with overweight (including obesity) (n = 5913)**

**Child lifestyle behaviors**	**Crude model**	**Model 1** ^*^	**Model 2** ^**^	Model 3***
	**OR (95 CI%)**	**OR (95% CI)**	**OR (95% CI)**	**OR (95% CI)**
TV viewing (≥2 hrs/d)	**1.75 (1.47,2.08)** ^**‡**^	1.13 (0.94,1.35)^¥^	1.08 (0.89,1.31)	-
Computer game (≥1 hr/d)	1.22 (0.87,1.69)	0.88 (0.63,1.24)	0.86 (0.60,1.24)	-
Outdoor play (<1 hr/d)	**1.40 (1.19,1.64)** ^**‡**^	**1.23 (1.06,1.44)** ^**‡**^	1.16 (0.97,1.39)^¥^	1.16 (0.97,1.39)^¥^
Sport participation (no)	**1.29 (1.11,1.50)** ^**‡**^	1.09 (0.93,1.27)	1.08 (0.91,1.28)	-
Active transport (<5 d/week)	**0.72 (0.59,0.87)** ^**‡**^	0.92 (0.76,1.10)	0.94 (0.78,1.13)	-

Table is based on imputed dataset. ^¥^p value <0.20, ^†^p value <0.10; ^‡^p value <0.05. Values in bold indicate statistical significance (p < 0.05).

Values represent odds ratios and 95% confidence intervals derived from multiple logistic regression analyses.

^*^Adjusted for socio-demographic factors: child’s sex, child’s age, child’s ethnicity, maternal educational level, household income, and maternal employment status.

**Additionally adjusted for family lifestyle factors: child’s breakfast skipping, consumption of high-calorie snacks, consumption of sugar-containing beverages, maternal BMI, and paternal BMI.

***Additionally adjusted for other sedentary behaviors and physical activity behaviors.

**Table 3:** **Associations of sedentary behaviors and physical activity behaviors with BMI SDS (n = 5913)**

**Child lifestyle behaviors**	**Crude model**	Model 1*	Model 2**	Model 3***
	**β (95% CI)**	**β (95% CI)**	**β (95% CI)**	**β (95% CI)**
TV viewing (≥2 hrs/d)	**0.19 (0.12,0.27)** ^**‡**^	0.03 (−0.05,0.11)	0.01 (−0.07,0.09)	-
Computer game (≥1 hr/d)	**0.12 (0.01,0.24)** ^**‡**^	−0.01 (−0.12,0.11)	−0.01 (−0.12,0.09)	-
Outdoor play (<1 hr/d)	**0.12 (0.06,0.18)** ^**‡**^	**0.07 (0.02,0.13)** ^**‡**^	0.04 (−0.02,0.11)^¥^	0.04 (−0.02,0.11)^¥^
Sport participation (no)	0.04 (−0.02,0.09)^¥^	−0.04 (−0.09,0.02)^¥^	−0.04 (−0.09,0.01)^¥^	−0.04 (−0.09,0.01)^¥^
Active transport (<5 d/week)	**−0.09 (−0.15,-0.03)** ^**‡**^	−0.01 (−0.07,0.05)	0.00 (−0.05,0.05)	-

Table is based on imputed dataset. ^¥^p value <0.20, ^†^p value <0.10; ^‡^p value <0.05. Values in bold indicate statistical significance (p < 0.05).

Values represent beta’s and 95% confidence intervals derived from multiple logistic regression analyses.

^*^Adjusted for socio-demographic factors: child’s ethnicity, maternal educational level, household income, and maternal employment status.

**Additionally adjusted for family lifestyle factors: child’s breakfast skipping, consumption of high-calorie snacks, consumption of sugar-containing beverages, maternal BMI, and paternal BMI.

***Additionally adjusted for other sedentary behaviors and physical activity behaviors.

**Table 4:** **Associations of sedentary behaviors and physical activity behaviors with percent fat mass (%) (n = 5913)**

**Child lifestyle behaviors**	**Crude model**	Model 1*	Model 2**	Model 3***
	**β (95% CI)**	**β (95% CI)**	**β (95% CI)**	**β (95% CI)**
TV viewing (≥2 hrs/d)	**1.42 (0.98,1.86)** ^**‡**^	**0.43 (0.02,0.84)** ^**‡**^	0.31 (−0.08,0.70)^¥^	0.29 (−0.11,0.68)^¥^
Computer game (≥1 hr/d)	0.19 (−0.64,1.02)	0.10 (−0.58,0.78)	0.04 (−0.59,0.68)	-
Outdoor play (<1 hr/d)	**0.75 (0.23,1.27)** ^**‡**^	0.35 (−0.02,0.72)^†^	0.20 (−0.20,0.61)	-
Sport participation (no)	**0.94 (0.58,1.29)** ^**‡**^	**0.62 (0.33,0.91)** ^**‡**^	**0.59 (0.31,0.87)** ^**‡**^	**0.58 (0.30,0.87)** ^**‡**^
Active transport (<5 d/week)	**−0.68 (−1.07,-0.28)** ^**‡**^	−0.02 (−0.34,0.30)	0.02 (−0.27,0.31)	-

Table is based on imputed dataset. ^¥^p value <0.20, ^†^p value <0.10; ^‡^p value <0.05. Values in bold indicate statistical significance (p < 0.05).

Values represent beta’s and 95% confidence intervals derived from multiple logistic regression analyses.

^*^Adjusted for socio-demographic factors: child’s sex, child’s age, child’s height, child’s ethnicity, maternal educational level, household income, and maternal employment status.

**Additionally adjusted for family lifestyle factors: child’s breakfast skipping, consumption of high-calorie snacks, consumption of sugar-containing beverages, maternal BMI, and paternal BMI.

***Additionally adjusted for other sedentary behaviors and physical activity behaviors.

**Table 5:** **Associations of lifestyle behaviors with overweight and obesity (n = 5913)**

	**Overweight (excluding obesity)**			
	**(n = 782)**			
**Child lifestyle behaviors**	**Crude model**	Model 1*	Model 2**	Model 3***
	**OR (95 CI%)**	**OR (95 CI%)**	**OR (95%CI)**	**OR (95%CI)**
TV viewing (≥2 hrs/d)	**1.60 (1.34,1.91)** ^**‡**^	1.13 (0.92,1.37)	1.09 (0.89,1.34)	-
Computer game (≥1 hr/d)	1.09 (0.72,1.64)	0.83 (0.54,1.28)	0.82 (0.53,1.28)	-
Outdoor play (<1 hr/d)	**1.38 (1.16,1.66)** ^**‡**^	**1.26 (1.06,1.49)** ^**‡**^	1.20 (0.99,1.45)^†^	1.20 (0.99,1.45)^†^
Sport participation (no)	**1.29 (1.10,1.52)** ^**‡**^	1.12 (0.95,1.33)^¥^	1.12 (0.94,1.33)	-
Active transport (<5 d/week)	**0.74 (0.60,0.92)** ^**‡**^	0.92 (0.74,1.14)	0.93 (0.74,1.15)	-
	**Obesity (excluding overweight)**			
	**(n = 261)**			
**Child lifestyle behaviors**	**Crude model**	Model 1*	Model 2**	Model 3***
	**OR (95 CI%)**	**OR (95 CI%)**	**OR (95% CI)**	**OR (95% CI)**
TV viewing (≥2 hrs/d)	**2.23 (1.60,3.12)** ^**‡**^	1.13 (0.80,1.59)	1.06 (0.71,1.57)	-
Computer game (≥1 hr/d)	1.61 (0.98,2.64)^†^	1.01 (0.59,1.72)	0.99 (0.56,1.78)	-
Outdoor play (<1 hr/d)	**1.44 (1.08,1.92)** ^**‡**^	1.17 (0.86,1.59)	1.05 (0.73,1.51)	-
Sport participation (no)	1.29 (0.98,1.68)^†^	0.96 (0.72,1.28)	0.93 (0.67,1.28)	-
Active transport (<5 d/week)	**0.65 (0.49,0.85)** ^**‡**^	1.05 (0.78,1.41)	1.06 (0.78,1.43)	-

Table is based on imputed dataset. ^¥^p value <0.20, ^†^p value <0.10; ^‡^p value <0.05. Values in bold indicate statistical significance (p < 0.05).

Values represent odds ratios and 95% confidence intervals derived from multivariable multinomial regression analyses (reference category is normal weight).

^*^Adjusted for socio-demographic factors: child’s sex, child’s age, child’s ethnicity, maternal educational level, household income, and maternal employment status.

**Additionally adjusted for family lifestyle factors: child’s breakfast skipping, consumption of high-calorie snacks, consumption of sugar-containing beverages, maternal BMI, and paternal BMI.

***Additionally adjusted for other sedentary behaviors and physical activity behaviors.

## Discussion

This study aimed to assess the independent associations of key sedentary and physical activity behaviors with three different indicators of body fat, including percent fat mass, BMI SDS, and weight status. Sports participation was independently inversely associated with percent fat mass, but not with BMI SDS or weight status. No other independent associations were observed.

### Television viewing

Although television viewing was positively associated with all indicators of body fat in the unadjusted models, the associations with weight status and BMI SDS disappeared after correction for socio-demographic factors such as family socioeconomic position and child’s ethnicity. Analyses using percent fat mass, the most accurate measure of body fatness, showed that the association with television viewing remained significant after adjustment for socio-demographic factors but disappeared after adjustment for family lifestyle factors such as children’s dietary behaviors and parental BMI. These results contradicts previous research that has shown consistent cross-sectional and longitudinal associations between children’s television viewing and risk of overweight and obesity [7]. As an explanation for our results, we hypothesize that children of this age may only start to show excessive weight gain after extended exposure to high levels of television viewing. Alternatively, for the purpose of this study, we defined family lifestyle factors as potential confounders in the associations between each of the sedentary behaviors and physical activity behaviors and the three outcomes. However, previous studies have suggested that unhealthy dietary behaviors (i.e., increased consumption of snacks and sugar-sweetened beverages during and following screen time) may mediate part of the effects of television viewing on childhood obesity [35-37]. Therefore, if we assume that children’s consumption of snacks and sugar-sweetened beverages are part of the causal pathway linking television viewing with children’s body fatness, television viewing may also be considered a modifiable risk factor of children’s percent fat mass.

### Computer game use

Computer game use was associated with BMI SDS in the crude model only. No other associations were observed. Similarly to television viewing, longer exposure to this sedentary activity may be necessary to detect any effects on children’s body fat. Also, computer game use included active video games and higher energy expenditure during such activities may not pose a risk for weight gain [7]. Alternatively, the lack of variation in this variable (i.e., a vast majority of children uses computers <1 hour/day) might have led to a lack of power to detect an association.

### Sports participation

No independent associations were found between sports participation and BMI SDS or weight status; however, a significant inverse association was found between sports participation and percent fat mass, even after adjustment for socio-demographic factors, family lifestyle factors, and other sedentary behaviors and physical activity behaviors. Previous studies on the associations between children’s physical activity intensity and adiposity have shown that moderate-to-vigorous physical activity, vigorous physical activity in particular, is associated with decreased adiposity [12,38-40]. High levels of physical activity are most often reached during sports activities [41], and the examples stated in our question assessing sports participation (e.g., gymnastics, tennis, and soccer) can be considered moderate-to-vigorous intense activities in this age group [42]. Since percent fat mass is a more accurate measure of body fatness compared to BMI or weight status, associations may be more easily detected with this indicator. Contrary to the present study, Drenowatz et al. did find an inverse association between sports participation and the odds of being overweight [43]. This discrepancy in findings may be explained by the age difference between their study (8 year old children) and the present study (6 year old children); older children may have spent more years participating in sports, which may result in demonstrable effects on weight status. Furthermore, children aged 8 years may have a higher weekly frequency of sports participation, or may engage in higher intensity levels during sports activities compared with younger children [41,44]. Alternatively, sports participation may be an indicator of an overall healthy lifestyle. However, the association between sports participation and percent fat mass remained significant after adjustment for other lifestyle behaviors, including television viewing and important dietary behaviors. Due to the many comparisons made in this study, it is also possible that spurious associations may have occurred as a result of multiple testing [45]. However, since the association was highly significant (p = 0.000), this explanation is rather unlikely.

### Outdoor play

Outdoor play was not independently associated with any of the outcome measures. These findings are consistent with a previous study conducted among 5–6 year-old Dutch children [28]. For 6-year-old children, physical activity guidelines state that children should be active for at least 60 minutes per day at a moderate to vigorous level in order to convey beneficial health effects [23-25]. Since we did not have any information on actual physical activity levels during outdoor play, it is possible that children were not physically active enough during outdoor play to find any effects on children’s body fat [41]. A recent study among 4- to 5-year-old preschool children suggests that children playing outdoor spend under 21% of time in moderate to vigorous physical activity [46]. Also, similar to sedentary activities, it may be that more extended exposure to low levels of physical activity is needed before any effects become visible.

### Active transport to and from school

In the unadjusted models, children using active transport less than 5 days per week were significantly less likely to be overweight, and had significantly lower BMI SDS and percent fat mass. These associations disappeared after taking into account socio-demographic characteristics of the child and the family. Indeed, additional analyses showed that native Dutch children and children from high socioeconomic families, children shown to be at decreased risk of overweight and obesity [47-49], were more likely use active transport less than 5 days per week compared to children of ethnic minority groups and children from lower socioeconomic families (data not shown). In addition, we did not account for distance to school. Children using active modes of transport may live closer to schools, and active transportation over a short distance may not be enough to change indicators of body fat [50]. These results are in concordance with the literature [51,52], also showing no associations between children’s active transport and body weight.

### Study strengths and limitations

Strengths of this study are the size of the study population and the measurement of important confounders. Furthermore, we were able to use percent fat mass as indicator of body fat, which is generally considered a highly accurate measure of body fat in young children [53]. In addition, the young age of the study population allowed us to assess modifiable risk factors of overweight and body fatness in a population that may still benefit from intervention programs. Several limitations should also be considered. First, the data for this study were cross-sectional, which precludes inferences about causality. Second, extended exposure to unfavorable lifestyle behaviors may be necessary before any effects on adiposity become measurable. Children with severe overweight (i.e., obesity) are likely to be exposed over a longer period of time. However, given that the multinomial logistic regression analyses yielded highly similar results, we have no indications that duration of exposure plays, or lack thereof, is a prominent explanation for the current results. Third, sedentary behaviors and physical activity behaviors were measured by parent-reported questionnaires. Although the questionnaires did not specifically refer to leisure time only, parents are likely to have reported on behaviors displayed outside school hours. As a consequence, time spent in sedentary behaviors and physical activity behaviors are likely to have been underestimated for weekdays. Also, the items measuring these behaviors were derived from questionnaires used by local and nation municipalities in The Netherlands and have not been tested for validity and reliability in children of this age [54]. Future studies should aim at incorporating objectively measured physical activity (i.e., by accelerometry and direct observation) in order to obtain more information about time spent in different activity levels and physical activity behaviors across the whole day. Finally, data on sedentary behaviors and physical activity behaviors were dichotomized according to current guidelines and recommendations [6,22-25]. This may have potentially led to a loss of information and statistical power to detect associations. We have re-analyzed our data using continuous exposure variables (data not shown). These analyses yielded highly similar results, with the exception of an additional significant independent association between outdoor play and percent body fat (beta = −0.18, p < 0.05).

## Conclusion

The results of this study indicate that sports participation is inversely associated with percent body fat among ethnically diverse 6-year-old children. More research in varied populations including objective measurements and longitudinal designs are needed to confirm these current results. In the meantime, health professionals should be aware that even at a young age attention should be given to children’s participation in organized sports or other high intensity physical activities.
